# Inflammatory Biomarkers are Correlated with Some Forms of Regressive Autism Spectrum Disorder

**DOI:** 10.3390/brainsci9120366

**Published:** 2019-12-11

**Authors:** Margherita Prosperi, Letizia Guiducci, Diego G. Peroni, Chiara Narducci, Melania Gaggini, Sara Calderoni, Raffaella Tancredi, Maria Aurora Morales, Amalia Gastaldelli, Filippo Muratori, Elisa Santocchi

**Affiliations:** 1 IRCCS Fondazione Stella Maris, Calambrone, 56128 Pisa, Italy; mprosperi@fsm.unipi.it (M.P.); scalderoni@fsm.unipi.it (S.C.); rtancredi@fsm.unipi.it (R.T.); fmuratori@fsm.unipi.it (F.M.); esantocchi@fsm.unipi.it (E.S.); 2 Department of Clinical and Experimental Medicine, University of Pisa, 56126 Pisa, Italy; diego.peroni@unipi.it; 3 Institute of Clinical Physiology, CNR, 56124 Pisa, Italy; letizia.guiducci@ifc.cnr.it (L.G.); mgaggini@ifc.cnr.it (M.G.); morales@ifc.cnr.it (M.A.M.); 4 Child and Adolescent Neuropsychiatry Unit, Department of Biomedical Science, University of Cagliari & “Antonio Cao” Paediatric Hospital, “G. Brotzu” Hospital trust, 09124 Cagliari, Italy; narducci.chiara@gmail.com

**Keywords:** autism spectrum disorder, regression, cytokines, PAI-1, neuroinflammation, gastrointestinal

## Abstract

*Background*: Several studies have tried to investigate the role of inflammatory biomarkers in Autism Spectrum Disorder (ASD), and their correlations with clinical phenotypes. Despite the growing research in this topic, existing data are mostly contradictory. *Methods*: Eighty-five ASD preschoolers were assessed for developmental level, adaptive functioning, gastrointestinal (GI), socio-communicative and psychopathological symptoms. Plasma levels of leptin, resistin, plasminogen activator inhibitor-1 (PAI-1), macrophage chemoattractant protein-1 (CCL2), tumor necrosis factor-alfa (TNF-α), and interleukin-6 (IL-6) were correlated with clinical scores and were compared among different ASD subgroups according to the presence or absence of: (i) GI symptoms, (ii) regressive onset of autism. *Results*: Proinflammatory cytokines (TNF-α, IL-6 and CCL2) were lower than those reported in previous studies in children with systemic inflammatory conditions. GI symptoms were not correlated with levels of inflammatory biomarkers except for resistin that was lower in ASD-GI children (*p* = 0.032). Resistin and PAI-1 levels were significantly higher in the group with “regression plus a developmental delay” onset (Reg+DD group) compared to groups without regression or with regression without a developmental delay (*p* < 0.01 for all). *Conclusions*: Our results did not highlight the presence of any systemic inflammatory state in ASD subjects neither disentangling children with/without GI symptoms. The Reg + DD group significantly differed from others in some plasmatic values, but these differences failed to discriminate the subgroups as possible distinct ASD endo-phenotypes.

## 1. Introduction

To date, the understanding of the underlying molecular mechanisms of some metabolic or neurological diseases and the deepening of knowledge on the role of inflammation in these disorders have radically changed our understanding of their etiology [1,2]. Alzheimer’s (AD) and Parkinson’s disease, type 1 and type 2 diabetes, and obesity are just some of the pathologies for which a well-defined role of inflammation has been identified, with consequent possible therapeutic implications [3,4]. For example, activated astrocytes and microglia are characteristically found in abundance near neurons and plaques in AD [5] and the block of the activation of insulin signaling receptors caused by the chronic exposure of pro-inflammatory mediators in β-cells of pancreatic islets has been evidenced in the pathogenesis of insulin resistance which underlies many metabolic diseases [6,7].

Recently, the contribution of immune dysregulation has been described as a common feature of the autism spectrum disorder (ASD), and alterations in circulating cytokine levels have been repeatedly reported [8,9]. ASD are neurodevelopmental disorders characterized by persistent social communication difficulties with concurrent restricted interests, repetitive activities and sensory abnormalities [10]. The etiopathogenesis of idiopathic ASD is complex and not yet fully elucidated, but it is widely recognized that genetic liability and environmental factors interact in producing early alteration of structural and functional brain development, responsible for ASD symptoms [11,12]. Despite a systematic review about proinflammatory markers in more than 3900 children and/or adolescents with neuropsychiatric disorders including ASD [13] found preliminary evidence for the role of inflammation and pro-inflammatory state in these conditions, until now conflicting and irreproducible findings have been detected in various studies.

Some authors have proposed interleukin (IL)-6, tumor necrosis factor-alpha (TNF)-α, and macrophage chemoattractant protein-1 (CCL2) as potentially involved in brain inflammation at least in a subgroup of subjects with ASD [14]. A recent meta-analysis of 25 studies revealed a higher concentration of pro-inflammatory cytokines interferon (IFN)-γ, IL-1β, IL-6, and TNF-α in children with ASD compared with controls [9]. Increased levels of IL-6 and IL-8 were found to be predictive biomarkers for ASD risk in a study analyzing circulating cytokine patterns from neonatal blood [15]. High levels of IL-6 in the brain could determine alterations of synapse formation, dendritic spine development, and neuronal circuit balance [16], while in plasma they have been associated with increased stereotypical behaviors and with regressive forms of ASD [17]. Conversely, TNF-α has a critical role in regulating synaptic strength and plasticity [18], and his levels have been positively correlated with ASD severity [19]. High CCL2 levels could be instead considered as a signal of microglia/astroglia activation [20], and have been associated with higher aberrant behavior scores and more impaired adaptive functioning [21].

Similarly, GI problems that frequently occur in ASD subjects seem to be caused by inappropriate immune activation and pro-inflammatory processes of the digestive tract [22]. It has been shown that the level of stress-responsive cytokines, like IL-6 and TNF-α, are increased both in ASD subjects [17] and in the general population in association to gastrointestinal (GI) symptoms [23,24], pointing to a link between peripheral inflammation and neuroinflammation. Particularly, high levels of TNF-α can influence the intestinal epithelial barrier possibly contributing to GI problems [25] and intestinal permeability, and also to ASD onset as recently suggests by the “leaky gut” hypothesis [26]. The myeloid dendritic cells, which produce among others TNF- α and IL-6, have been associated with increased GI symptoms in ASD as well as increased amygdalar volume and regressive autism [27]. More recently, other authors [22,28] did not confirm an association between the symptoms of the lower GI tract and levels of TNF-α or IL-6, however their levels were correlated with irritability, socialization and intelligence in ASD subjects.

Besides, a particular type of cytokines called adipokines seems to be implicated in the pathogenesis of inflammatory central nervous system (CNS) disorders and ASD [29] despite the findings obtained so far are mostly controversial. Adipokines, or adipocytokines, are active proteins secreted by white adipose tissue with functions similar to hormones in inter-organ communication [30] and their dysregulation has been implicated in obesity, type 2 diabetes, cardiovascular disease and recently, in peripheral tissue insulin resistance and inflammation [31]. Leptin, adiponectin and resistin are the only three molecules that belong exclusively to the class of adipokines and they have been studied in a limited number of researches concerning autism. Increased levels of leptin, decreased levels of resistin and a negative correlation between the levels of adiponectin and the severity of social impairment were found in the plasma of ASD subjects vs. controls [29]. Previously, Blardi et al. [32,33] found higher levels of leptin in patients with Rett syndrome in comparison with healthy female subjects, as reported by Ashwood et al. [34] in patients with autism compared to typically developing controls. Leptin dysregulation has been proposed as a mechanism of psychopathology associated with mental health disorders [35], and elevated circulating leptin was consistently found in childhood neurodevelopmental disorders including ASD [34].

Resistin has been implicated in the pathogenesis of several inflammatory CNS disorders [36] and its levels are related to immune changes in autistic subjects: it has been shown that proinflammatory cytokines may increase the expression of messenger-RNA resistin [37] with a positive correlation between increasing resistin levels and inflammatory serum cytokines [38]. A recent case-control study [39] found that resistin levels were increased in ASD subjects compared to healthy controls. To date, no studies have investigated differences in adipokines’ levels in ASD subjects with or without GI symptoms.

Distally regulated by some cytokines (i.e., IL-6, IL-1, and TNF-α), the plasminogen activator inhibitor-1 (PAI-1) seems to directly influence brain functions causing a neuronal dis-connectivity due to abnormal neuronal migration [40]. PAI-1 may regulate microglial migration and phagocytosis in an autocrine or paracrine manner playing an important role in the regulation of brain microglial activities in health and disease [41]. Moreover, his locus in human maps very close to or within a region in chromosome 7 linked to autism. No association was found between the presence of ASD and a particular polymorphism of the PAI-1 gene promoter that affects the PAI-1 plasma levels [40].

This pilot study aims (i) to investigate the plasmatic levels of several proinflammatory molecules (TNF-α, IL-6, CCL2, leptin, resistin, and PAI-1) in preschoolers with ASD; (ii) to explore the correlation between their plasmatic levels and behavioral profiles in preschoolers with ASD to detect possible specific subgroups within the ASD heterogeneity.

## 2. Materials and Methods

### 2.1. Participants

A total of 85 ASD preschoolers were included in the study and recruited from November 2015 to February 2018 at the ASD Unit of the IRCCS Stella Maris Foundation (Pisa, Italy), a tertiary care university hospital during a clinical trial on the efficacy of probiotic supplementation in ASD preschoolers [42]. In the present study baseline clinical and biochemical data of recruited subjects were investigated.

ASD diagnosis was performed according to Diagnostic and Statistical Manual of Mental Disorders (DSM)-5 [10] criteria by a multidisciplinary team. Exclusion criteria were brain anomalies; neurological syndromes/focal neurological signs; anamnesis of birth asphyxia, severe premature birth/perinatal injuries; epilepsy; significant sensory impairment; diagnosis of organic GI disorder or coeliac disease; special diets; recent any-known infections that could influence circulating cytokines.

All children had a comprehensive evaluation including Autism Diagnostic Observation Schedule-2 (ADOS-2) [43], Griffiths Mental Development Scales-Extended Revised (GMDS-ER) [44], Vineland Adaptive Behavior Scales-Second edition (VABS-II) [45], Child Behavior CheckList 1.5-5 (CBCL 1.5-5) [46], Repetitive Behavior Scale-Revised (RBS-R) [47], Social Communication Questionnaire (SCQ) [48]. The “Overall Level of Non-Echoed Spoken Language” item (A1 score) of the ADOS-2 was used to differentiate non-verbal (those with absent language or less than 5 words) from verbal children: 39 participants (46%) were verbal and 46 (54%) were non-verbal. Information about pharmacological treatments and food supplements in the previous 3 months were collected: parents reported an acute or episodic administration of antibiotics (28.2%), probiotics (8.2%), NSAIDs or paracetamol (14.1%), steroids (8.2%), other drugs without effects on GI symptoms (36.5%), and a chronic administration of osmotic laxatives (12.9%). None of the enrolled subjects used psychotropic drugs.

The demographic and clinical characteristics and a complete description of the tools of all participants and in no-verbal vs. verbal groups are reported in Table 1.

To evaluate the presence of GI symptoms we used a modified version of the GI Severity Index (GSI [49]) splitting the subjects into two groups (GI vs. No-GI). GSI is a score designed to identify signs and symptoms of GI distress commonly reported by parents of children with ASD including nine variables, the first six exploring specific GI symptoms (constipation, diarrhea, stool consistency, stool smell, flatulence, abdominal pain) and three exploring unexplained daytime irritability, night-time awakening, and abdominal tenderness. A total score of 4 and above (with at least 3 score points from the first six items) was considered clinically significant for the classification of a subject within the GI group.

Moreover, all preschoolers were divided into regressive or non-regressive (early-onset -EO-ASD-) autism based on the presence/absence of a history of loss of competences such as language or social competences [50]; children belonging to regressive group were further divided in those with regression plus a previous developmental delay (Reg + DD) and those without a previous developmental delay (Reg − DD). According to Kern et al. [51], “regression *plus* developmental delay” was defined as a significant lag in the appearance of normal developmental milestones with a later loss of previously acquired skills.

This study was carried out according to the standards for good ethical practice and with the guidelines of the Declaration of Helsinki. The study protocol was approved by the Pediatric Ethics Committee of the Tuscany Region (Approval Number: 126/2014). Written informed consent from a parent/guardian of each participant was obtained.

### 2.2. Blood Sample Collection

A fasting blood sample (3 mL for each child) was collected in Ethylenediamine tetraacetic acid (EDTA) tube to perform the cytokines quantitative analysis. We didn’t use pain patch before the sampling. Each tube was centrifuged for 10 min at 3500 rpm and all the plasma samples were stored at −80 °C until required the bio-humoral investigations

### 2.3. Cytokine Analysis

The cytokines were measured directly in the plasma through specific immunometric tests (MILLIPLEX MAP, human-magnetic bead panel, Millipore Corporation, Billerica, MA, USA) using an integrated multi-analyte detection platform (high-throughput technology Magpix system, Luminex xMAP technology, Luminex, Austin, TX, USA) 

Each sample was analyzed in duplicate. In each one, a sample was analyzed as a quality control. Inter-assay variability was evaluated using two samples at different concentrations and was <10%.

### 2.4. Statistical Analysis

Descriptive statistics were computed for selected demographic variables across diagnostic groups. Contingency tables were used to perform the frequency analysis. Since the molecule’s values were not normally distributed, we used log-transformed values with parametric statistic tests and non-parametric tests to compare GI vs. No-GI subjects (Mann-Whitney test) and to compare EO ASD vs. Reg-DD vs. Reg + DD (Kruskall-Wallis test) for all the selected molecules.

Correlation and regression analysis were computed to study the relationship between the molecules and the identified clinical parameters. Findings with *p* value <0.05 were considered significant. StatView software (version 5.0.1; SAS Institute, Abacus Concept Inc., Berkeley, CA, USA) was used for data analyses. To discriminate different subgroups of ASD children based on biomarker levels, we performed Principal Component Analysis (PCA) using as correlated variables: sex, BMI, age, and cytokine levels (TNFa, IL6, CCL2, leptin, resistin and PAI 1). After log transformation and auto scaling (e.g., mean-centered and divided by standard deviation of each variable) PCA was performed using MetaboAnalystR 1.0.3 (Xia Lab, McGill University, Montreal, Canada). We checked quality control of samples using PCA that allowed us to label the 85 samples as outlier so it was excluded from downstream analysis.

## 3. Results

Thirty children (35%) were in the GI group and 55 (65%) in the No-GI group. Among the 30 GI subjects, 20 children (67%) were in the non-verbal group, whereas among the 55 No-GI, 26 children (47%) were in the non-verbal group. No statistically significant differences were found in the prevalence of GI subjects between verbal and non-verbal groups (*p* = 0.086). As concerns sex distribution, no differences were found in the prevalence of females in GI versus No-GI groups neither verbal versus non-verbal groups (*p* = 0.560 and *p* = 0.804, respectively).

As concerns clinical variables, there were no significant differences between the GI and the No-GI groups, with the exception of the Global Score of the RBS-R (60.24 ± 20.77 vs. 38.12 ± 27.06; *p* = 0.0016), the Internalizing and Total problem scores of the CBCL (all significantly higher in the GI group than in the No-GI group: 67.48 ± 7.80 vs. 62.06 ± 9.04, *p* = 0.0065 and 65.35 ± 10.02 vs. 60.62 ± 10.30, *p* = 0.0469, respectively), and of the Communication and Daily Living adaptive scores of the VABS (significantly higher in the No-GI group than in the GI group: 45.47 ± 15.22 vs. 54.46 ± 18.80 *p* = 0.0274 and 61.13 ± 14.29 vs. 69.07 ± 17.51 *p* = 0.0365, respectively).

As concerns proinflammatory cytokines levels, the single and the mean values in the total sample and in each subgroup are reported in Table 2. We did not find significant differences in the levels of plasmatic cytokines between GI and No-GI group except for resistin levels (*p* = 0.032). No difference in plasma biomarker levels was found between non-verbal and verbal groups.

Regarding the onset of autism, the mean values of cytokines were not statistically significant different between EO-ASD and regressive subgroups. Nevertheless, comparing cytokines levels in the EO-ASD subgroup with the two types of regressive preschoolers (with and without DD), resistin and PAI-1 levels were statistically significant higher in the Reg + DD group than in the other two groups, the EO-ASD and the Reg-DD ones (*p* < 0.01 for all). 

Finally, after the correlation analysis between each molecule and all the clinical parameters, CCL2 levels negatively correlated with CBCL1.5-5 Internalizing and Total problems (*p* = 0.0003, *R* = 0.383 and *p* = 0.013, *R* = −0.272, respectively) and with RBS-R total scores (*p* = 0.05, *R* = 0.21), and positively correlated with VABS-II Motor Skills (*p* = 0.019, *R* = 0.25). TNF-α and PAI-1 levels negatively correlated with age (*p* = 0.0005, *R* = −0.37 and *p* = 0.024, *R* = −0.25, respectively); Leptin levels positively correlated with Body Mass Index (*p* = 0.002, *R* = 0.34) and negatively correlated with CBCL1.5-5 Internalizing problems (*p* = 0.0086, *R* = −0.29).

**Figure 1:**
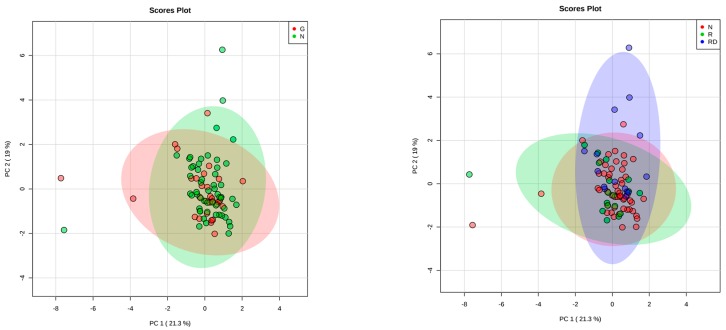
In the left plot, the Principal Component Analysis in gastrointestinal (red) and non-gastrointestinal subjects (green) is presented; in the right plot the PCA based on the ASD onset is presented: subjects with early-onset in red, regression without a previous developmental delay in green, regression plus a previous developmental delay in blue.

PCA analysis showed that the variability within the components explains the subdivision in clusters (No-GI vs. GI and EO-ASD vs. Reg − DD vs. Reg + DD) with a low percentage (PC1 = 21.3% and PC2 = 19.0%), indicating that the two and three groups respectively are not partially separated but overlapped (Figure 1).

## 4. Discussion

Our study fits within the complexity and the heterogeneity of studies that examine inflammation and immunity dysfunctions in ASD subjects, moving the field forward into the investigation of biological biomarkers to discriminate possible endophenotypes. The narrow age range considered, the detailed clinical characterization with specific and gold-standard tools for ASD evaluation, and an enough large sample represent the strengths of the study.

First, we found that the single and the mean values of our cytokines were lower than those expected in subjects with systemic inflammation [52,53,54]. These findings are in agreement with a part of the literature on this topic in which there is an absence of any atypical profile in the expression of relevant plasma cytokines both within ASD subjects and in comparison with TD children [55]. Regarding plasmatic cytokines, it should be highlighted that in literature the reference values and in particular those relating to the pediatric age, to date, are not definitively characterized. Despite our attempt to define specific subgroups based on cytokines levels and anthropometric measures using PCA, in our sample different endophenotypes were not identified. These results exclude the possibility that bringing all cases together in a single ASD group could have hidden significant results in one specific subgroup of preschoolers, as previously hypothesized [56,57]. Consequently, our findings do not support the use of anti-inflammatory therapies in ASD children, not even in a specific subgroup of ASD subjects as previously suggested [58].

Second, we did not observe significant differences in the levels of circulating cytokines between GI and No-GI ASD children, except for resistin. Notably, there is too scant relevant research on this topic in ASD subjects [29,39] to draw valid and accurate conclusions. Thus, the role of adipokines needs further studies, in particular, in correlation with GI symptomatology in ASD considering also the influence of fat mass in plasmatic levels of adipokines. These findings suggest that the frequently reported GI symptoms in ASD children seem to be independent from an inflammatory condition, confirming a not yet clarified meaning of these symptoms [59]. Previously, only a modest relationship between GI symptoms and TNF-α levels was detected [17,28], in one case [28] in significantly older subjects (school-aged children and adolescents) than ours. Specifically, when Ferguson et al. [28] considered only inferior GI symptoms (as we did) they did not identify any statistically significant correlations, in line with the findings that TNF-α levels are independent from the presence of GI symptoms [22,60]. Some authors [61,62,63,64] have measured the presence of cytokine-producing cells directly in the bowel of subjects with ASD, and found a local high level of these cells in patients with GI symptoms, supporting a local role of the inflammatory cytokines in altering intestinal epithelial barrier and thus in contributing to GI symptoms. Besides, we confirm our previous findings showing that ASD subjects with GI problems have worse clinical functioning than ASD subjects without GI problems, independently from the severity of autistic symptoms [65].

We did not find any significant correlations between the basal levels of TNF-α and IL-6 and the autistic features of the total sample, similarly to some investigations [56,66] and in contrast to others [17,28,67,68]. Moreover, we found a positive, though weak, correlation between CCL2 and better functioning of children, evaluated with the CBCL1.5-5, RBS-R and VABS-II, in contrast with studies reporting a significant correlation between higher CCL2 plasmatic levels and more severe impairment of the autistic condition [21,57,69]. Further studies are necessary to disentangle the controversial findings on the possible role of some cytokines as sensible markers of the impairment in ASD children.

Third, we found that the group with regression plus developmental delay prior to the onset of ASD (16.5% of the sample) was significantly different from the rest of the sample as far as the higher plasmatic levels of resistin and PAI-1. We could suggest that Reg + DD children represent a specific subgroup with a definite biological profile and a specific clinical feature. However, using the PCA method, we did not identify the Reg + DD group as a particular cluster of patients, making the individuation of a specific endophenotype unlikely in this sample. Future studies are needed to retest the robustness of these findings before we can consider them as reliable.

In addition, we did not identify any significant correlation between the levels of cytokines and the presence or absence of a regression of skills prior to the onset of autism. This result is in accordance with the majority of similar investigations, but in contrast with others where an association, although weak, between regressive autism and TNF-α [70], or lower plasma leptin levels [34] was found. Previous studies detected higher basal plasmatic levels of IL-1β [17,69], IL-5 [69], IL-17 [69] and higher levels of neural cell adhesion molecule (NCAM) [55]—a molecule playing a role in cell–cell adhesion, neurite outgrowth, synaptic plasticity, learning and memory—in subjects with a regression of skills prior to the onset of autism. More broadly, ASD subjects with regression have been repeatedly identified as different in pathophysiological findings from ASD subjects without regression both in terms of neuroanatomy [71], and EEG patterns [72]. However, there is an urgent need to study the clinical regression in ASD, since a clear understanding of the definition, prevalence, etiopathogenesis, age of onset, and outcome profiles of this complex phenomenon is far from being concluded [73,74].

### Limitations

We must consider this study as a pilot investigation with several limitations. Compared to other authors who have measured a series of pro-inflammatory cytokines in ASD subjects [22], we focused our analysis on six cytokines, so limiting the possible range of our results. The changes in the expression of cytokines due to subjects’ age [75] have already been described, and we cannot exclude that our results on inflammatory markers could be age-specific; in addition, we have to consider that sex, sleep-wake cycle and the percentage of fat mass, which could increase that variability [76,77] representing possible interfering factors, have not been assessed in this study. Moreover, the low number of females within our sample of preschoolers with ASD did not allow us to accurately investigate possible sex differences in pro-inflammatory cytokine profiles.

## 5. Conclusions

Despite the above-mentioned limitations and the existing controversies within the studies about the role of cytokines in ASD and the extreme variability of their findings, our study finds no evidence of the presence of inflammatory condition in ASD subjects, except for resistin. Our findings do not support the use of anti-inflammatory therapies in ASD children, and paves the way for the search of alternative hypotheses for the etiology of GI symptoms in subjects with ASD. Despite our findings showed a specific plasmatic cytokine profile in ASD children with a history of a regressive way of onset within a previous developmental delay, the specific endophenotype for these subjects has not been identified.

### Ethics Approval and Consent to Participate

The study protocol was approved by the Pediatric Ethics Committee of Tuscany Region (Protocol Number: 126/2014), with written informed consent obtained from a parent/guardian of each participant. The study was conducted following the 1964 Declaration of Helsinki and its later amendments, and the International Conference on Harmonisation Guidelines for Good Clinical Practice.

## Figures and Tables

**Table 1:** Clinical characteristics of the total sample and in non-verbal vs. verbal group.

	Total Sample (*n* = 85; 100%)	Non-Verbal (*n* = 46; 54%)	Verbal (*n* = 39; 46%)	*p*	*p*, Age-adjusted
**AGE (years) mean ± SD**	4.14 ± 1.08 (range 2.18–6.11)	3.74 ± 0.96	4.62 ± 1.02	<0.0001	-
**MALES**	71 (83.5%)	38 (44.7%)	33 (38.8%)	NS	-
**FEMALES**	14 (16.5%)	8 (9.4%)	6 (7.1%)		-
**Weight (Kg)**	17.70 ± 3.09	17.06 ± 3.1	18.56 ± 2.89	0.026	NS
**BMI (Kg/m^2^)**	15.95 ± 1.66 (range 12.75–21.43)	16.07 ± 1.74	15.82 ± 1.54	NS	NS
**Head Circumference (cm)**	51.21 ± 1.69 (range 55–46)	51.31 ± 1.83	51.09 ± 1.54	NS	NS
**ADOS-2 CSS Score ^a^ (mean ± SD)**					
Social Affect	6.43 ± 2.05	7.06 ± 1.73	5.74 ± 2.09	0.002	n.a.*
Restricted and Repetitive Behaviors	8.23 ± 1.46	8.56 ± 1.36	7.95 ± 1.50	NS	n.a.*
Total	7.05 ± 1.85	7.72 ± 1.50	6.41 ± 1.90	0.0007	n.a.*
**GMDS-ER ^b^ (mean ± SD)**					
Performance Quotients	70.75 ± 23.33	61.47 ± 19.42	78.75 ± 23.73	0.0018	n.a.*
**VABS-II ^c^ (mean ± SD) Quotients**					
Communication	50.86 ± 17.79	40.76 ± 10.24	63.69 ± 17.43	<0.0001	n.a.*
Daily Living	66.56 ± 17.07	60.46 ± 13.14	73.13 ± 18.16	0.0002	n.a.*
Socialization	63.55 ± 15.02	57.35 ± 10.36	71.15 ± 16.53	<0.0001	n.a.*
Motor Skills	71.88 ± 17.85	70.89 ± 17.64	75.46 ± 16.75	NS	n.a.*
Composite Score	59.40 ± 19.53	52.96 ± 17.52	67.23 ± 19.61	0.0007	n.a.*
**CBCL 1.5-5 ^d^ T-score (mean ± SD)**					
Internalizing Problems	63.85 ± 9.06	64.98 ± 8.30	62.72 ± 9.64	NS	NS
Externalizing Problems	57.10 ± 9.09	56.71 ± 8.68	57.20 ± 9.55	NS	NS
Total Problems	62.28 ± 10.51	62.73 ± 10.68	61.69 ± 10.24	NS	NS
Sleep Problems	58.21 ± 9.11	59.62 ± 10.45	56.44 ± 6.83	NS	NS
Attention Problems	64.15 ± 8.21	64.66 ± 8.47	63.56 ± 7.98	NS	NS
Aggressive Behavior	56.58 ± 7.13	56.27 ± 5.93	56.95 ± 8.38	NS	NS
Attention Deficit/Hyperactivity Problems	59.31 ± 7.70	59.58 ± 7.51	59.00 ± 8.00	NS	NS
**RBS-R ^e^ (mean ± SD)**					
Total Score	19.87 ± 13.87	17.67 ± 10.25	22.41 ± 16.91	NS	NS
Total Endorsed Score	12.76 ± 7.27	11.91 ± 5.88	13.74 ± 8.58	NS	NS
Low Index	9.44 ± 6.07	9.33 ± 5.67	9.56 ± 5.59	NS	NS
High Index	10.25 ± 9.91	8.09 ± 7.11	12.79 ± 12.04	0.028	0.028
**SCQ ^f^ (mean ± SD)**					
Total Score	14.98 ± 5.90	16.72 ± 5.28	13.18 ± 6.16	0.006	NS

^a^ ADOS-2 is a semi-structured assessment of communication, social interactions, play, imagination, and stereotyped or repetitive behaviors used as the gold standard tool for the diagnosis of ASD. Higher ADOS-2 CCS scores indicate greater severity of autism (range of possible scores for Total, Social Affect and Restricted and Repetitive Behavior is 1–10). ^b^ GMDS-ER are a developmental assessment procedure including five different subscales. We used the Performance subscale to measure the non-verbal skills of each child. Higher scores indicate greater non-verbal abilities. Scores around 100 indicate normal non-verbal skills; scores below 70 indicate a developmental delay of non-verbal skills. ^c^ VABS-II is a parent interview that assesses adaptive functioning in different daily skills. Higher scores indicate greater adaptive skills, scores around 100 indicate a normal adaptive functioning and scores below 70 indicate a delay with respect to age. ^d^ CBCL 1.5-5 is a parent-report questionnaire that includes 100 statements about the child’s behaviors summarized into three summary scales (Internalizing, Externalizing and Total Problems). Besides, we have used the Aggressive Behavior, the Sleep Problems, the Attention Problems and the Attention Deficit/Hyperactivity (ADHD) Problems Scales of this tool as suggested by previous works on this argument. A T-score of 64 and above for summary scales, and a T-score of 70 and above for the other scales, are generally considered clinically significant. Values between 60 and 63 for summary scales, or between 65 and 69 for the other scales, identify a borderline clinical range. ^e^ RBS-R is a questionnaire completed by parents about the presence of a broad spectrum of repetitive behaviors. Higher scores indicate greater severity (range 0–114). A two-factor solution scoring of RBS-R was also adopted for this study: a Low-Level Index (composed of items pertaining to Stereotyped, and Self-Injurious subscales) and a High-Level Index (composed of items related to Compulsive, Ritualistic, Sameness and Restricted Interests Behaviors subscales). ^f^ SCQ is a 40-item parent-report screening measure evaluating the symptoms associated with ASD. We used the form “last three months”, completed by parents concerning the child’s last three months of life. Higher scores indicate greater severity (range 0–39) with a threshold of 15 compatible for a relevant impairment of social communication (some studies consider 9 in children younger than four years old). * Age adjustment is not due for ADOS-2 CCS, GMDS-ER and VABS-II since they are already standardized to compare subjects with different chronological ages. Abbreviations (alphabetic order): ADOS-2 Autism Diagnostic Observation Schedule-2; BMI Body Mass Index; CBCL 1.5-5 Child Behavior Checklist 1.5-5; CSS Calibrated Severity Score; GMDS-ER Griffiths Mental Development Scales-Extended Revised; n.a. not applicable; NS not significant; RBS-R Repetitive Behaviors Scale Revised; SCQ Social Communication Questionnaire; SD standard deviation; VABS-II Vineland Adaptive Behavior Scales-II.

**Table 2:** Comparisons between the cytokine levels in GI vs. No-GI groups, in EO ASD (a) vs. Reg-DD (b) vs. Reg+DD (c) subgroups and No-Verbal vs. Verbal groups. The mean levels of each cytokine in the total sample are also reported.

					a	b	c				
	Total Sample	No-GI 55 Subjects	GI 30 Subjects	*p* (No-GI vs. GI)	EO ASD	Reg − DD	Reg + DD	ANOVA *p* Value	NO VERBAL 46 Subjects	VERBAL 39 Subjects	*p* (No-V vs. V)
**N (%)**	85 (100)	55 (64.7%)	30 (35.3%)		57 (67.0)	14 (16.5)	14 (16.5)				
**TNF-α,** **m (SD) pg/mL** range 0.74–16.09	6.12 (2.40)	5.84 (2.01)	6.63 (2.95)	ns	6.09 (3.16)	6.76 (3.16)	5.56 (2.56)	ns	6.52 (2.57)	5.63 (2.50)	ns
**IL-6,** **m (SD) pg/mL** range 0.80–104.00	5.99 (16.17)	4.70 (13.83)	8.34 (19.80)	ns	5.74 (14.47)	10.82 (27.24)	2.18 (0.90)	ns	4.67 (6.96)	7.54 (22.69)	ns
**CCL2,** **m (SD) pg/mL** range 26.36–451.00	127.22 (58.81)	131.61 (66.86)	119.16 (39.90)	ns	126.85 (56.03)	135.10 (53.15)	120.84 (76.74)	ns	125.38 (53.73)	129.39 (64.95)	ns
**Leptin,** **m (SD) pg/mL** range 0.03–4.83	1.14 (0.89)	1.19 (0.96)	1.06 (0.76)	ns	1.26 (1.01)	0.96 (0.50)	0.88 (0.55)	ns	1.01 (0.80)	1.30 (0.97)	ns
**Resistin,** **m (SD) ng/mL** range 8.1–96.8	22.89 (13.63)	24.50 (14.37)	19.82 (11.74)	0.032	20.97 (10.45)	18.30 (9.68)	35.14 (20.75)	0.0003 (c > a) 0.0007 (c > b)	23.65 (15.78)	21.96 (10.60)	ns
**PAI-1,** **m (SD) ng/mL** range 5.5–91.2	26.04 (18.96)	27.52 (20.27)	23.24 (16.13)	ns	23.28 (12.74)	22.46 (14.04)	40.67 (33.68)	0.0018 (c > a) 0.0090 (c > b)	28.66 (22.86)	22.88 (12.32)	ns

ns: not significant; Abbreviations (in alphabetic order): ASD: Autism Spectrum Disorder; CCL2: Macrophage Chemoattractant Protein-1; EO ASD: early onset of ASD without a history of loss of competences; GI: gastrointestinal; IL-6: interleukin-6; PAI 1: Plasminogen Activator Inhibitor-1; Reg – DD: regression without a previous developmental delay; Reg + DD: regression with a previous developmental delay; SD: standard deviation; TNF-α: Tumor Necrosis Factor-alpha.

## Data Availability

The datasets generated and/or analyzed during the current study are not publicly available due to the privacy policy (containing information that could compromise research participant privacy/consent) but are available from the corresponding author on reasonable request and with permission of parents of the involved children.
